# Hypoglycemic effects of esculeoside A are mediated via activation of AMPK and upregulation of IRS-1

**DOI:** 10.1186/s12906-019-2543-3

**Published:** 2019-06-18

**Authors:** Ziming Yang, Li Zhang, Jinglei Liu, Fenglai Lu, Lei Wang, Yueyuan Chen, Dianpeng Li

**Affiliations:** 1 Guangxi Key Laboratory of Functional Phytochemicals Research and Utilization, Institute of Botany, Guangxi Zhuang Autonomous Region and Chinese Academy of Sciences, Guilin, 541006 China; 2 Guangxi Institute of Botany, Chinese Academy of Sciences, Road 85 Yanshan, Guilin, 541006 China

**Keywords:** Esculeoside a, *db/db* mice, AMPK, IRS-1, Liver glucose metabolism

## Abstract

**Background:**

Tomato fruit (*Lycopersicon esculentum* Mill.) has been suggested to be useful for the prevention of diabetes. Esculeoside A is the main saponin compounds in tomatoes. This study investigated the hypoglycemic effects and the underlying mechanism of esculeoside A in C57BLKS/Leprdb (*db/db*) mice.

**Methods:**

Wild-type C57BLKS (*db/dm*) mice were used in the *db/dm* mouse group and *db/db* mice were randomly divided into 2 groups: untreated and treated *db/db* mouse groups. Esculeoside A (100 mg/kg) was administered by gavage for 56 days to the treated *db/db* mouse group. Distilled water was administered to the *db/dm* mouse group and the untreated *db/db* mouse group. The blood and liver biochemical parameters and the expression of liver insulin signaling-related proteins were examined.

**Results:**

The results showed that esculeoside A reduced the fasting blood glucose (FBG) levels and improved the glucose tolerance. Further investigation revealed that hepatic protein expressions of total AMP-activated protein kinase (T-AMPK), phosphorylated AMP-activated protein kinase (p-AMPK), insulin receptor substrate-1 (IRS-1), and glucokinase (GCK) were significantly upregulated after esculeoside A treatment. In contrast, the hepatic protein expression of phosphoenolpyruvate carboxykinase (PEPCK) was significantly downregulated by esculeoside A treatment.

**Conclusion:**

These findings suggested that esculeoside A has a potential of alleviating the metabolic abnormalities in *db/db* mice via regulation of AMPK/IRS-1 pathway. Our findings supported a possible application of esculeoside A as a functional supplement for diabetes treatment.

## Background

Type 2 diabetes mellitus (T2DM) is a common metabolic disease worldwide. Amidst the worldwide epidemic of T2DM, 522 million people are estimated to suffer from T2DM by 2030 [1]. The increased incidence of T2DM has significantly increased the risk of associated complications, thereby reducing quality of life and increasing mortality. The patients with T2DM are prone to microvascular complications and macrovascular diseases, such as diabetic nephropathy, diabetic neuropathy, diabetic retinopathy, stroke, atherosclerosis, and hypertension [2, 3]. The basic pathogenesis of T2DM is characterized by hyperglycemia, relative impairment in insulin secretion, and insulin resistance [4]. In particular, insulin resistance is regarded as a major contributor in the occurrence and development of T2DM [5]. The liver is very important for metabolic homeostasis, and controls glucose utilization and production. It is a key organ for insulin activity. Insulin regulates lipogenesis and restrains gluconeogenesis in the liver. Insulin resistance leads to abnormalities in hepatic glucose output, and leads to hyperglycemia, which results in further worsening of the hepatic insulin insensitivity [6]. Insulin triggers series of signaling cascades at the cellular level, and insulin receptor substrate-1 (IRS-1) is crucial in this process. IRS-1 has also been linked to the treatment of hepatic insulin resistance [7]. Energy metabolism imbalance is a vital problem during T2DM. AMP-activated protein kinase (AMPK) is critical in regulating energy storage and utilization [8].

T2DM is often closely associated with dietary habits and lifestyle. With the socioeconomic development and changes in people’s diets, it is estimated that the prevalence of T2DM will increase tremendously over the next few decades. As a result, its high prevalence will cause great pressure on families and society, and it is important to find effective means to prevent the occurrence of T2DM.

Tomato is one of the most frequently consumed vegetables, and it has been suggested to be useful in preventing diabetes, obesity, coronary heart disease, hypertension, and other chronic diseases [9]. Studies have shown that lycopene, a component of tomato extract, can reduce blood sugar, improve lipid metabolism, and ameliorate diabetic nephropathy [10]. Esculeoside A was the first compound isolated from the cherry tomatoes (*Lycopersicon esculentum* Mill.); the quantity of esculeoside A was four times higher than that of lycopene in tomatoes [11]. Further investigation indicated that esculeoside A and its aglycone esculeogenin A could inhibit foam cell formation in vitro, reduce blood lipid levels, and inhibit the formation of atherosclerotic plaques in vivo [11].

Our previous studies have shown that the tomato saponin crude extract (TSCE) exhibited hypoglycemic effects in *db/db* mice (unpublished). To identify the bioactive components of TSCE, we previously analyzed the content of esculeoside A in cherry tomatoes and TSCE [12, 13]. Esculeoside A is a major constituent of TSCE (approximately 130 mg/g of TSCE), and accounts for 0.021% of dry weight of cherry tomatoes (*Lycopersicon esculentum*). Although previous studies have already shown that esculeoside A may alleviate lipid metabolic disorders [11], whether esculeoside A exhibits hypoglycemic effect during the treatment of diabetes is still unclear. The *db/db* mice represent a type of spontaneous obese diabetic mouse model [14], while the glucose and lipid metabolism disorders in these mice are consistent with human T2DM [15, 16]. In the present study, we analyzed the hypoglycemic effects of esculeoside A isolated from *Lycopersicon esculentum* in *db/db* mice, and investigated the possible mechanism of its action.

## Methods

### Chemicals and reagents

The serum total cholesterol (TC) and triglyceride (TG) kit were purchased from Changchun Huili Co., Ltd. (Changchun, China). ELISA kits for serum insulin (INS), tumor necrosis factor (TNF-α), interleukin-6 (IL-6), and interleukin-1β; ELISA kits for tissue TNF-α, IL-6, IL-1β; and kits for superoxide dismutase (SOD), malondialdehyde (MDA), serum alanine aminotransferases (ALT), and aspartate aminotransferases (AST) were purchased from Nanjing Jiancheng Bioengineering Institute (Nanjing, China). The tissue TC and TG assay kits were purchased from Beijing ApplyGen Technologies Inc. (Beijing, China). The total protein extraction kit was purchased from Nanjing KeyGen Biological Technology Co., Ltd. (Nanjing, China). The BCA protein quantitation kit was purchased from Biyuntian Co., Ltd. (Wuhan, China). The primary anti-p-AMPK (sc-98,485), anti-T-AMPK (sc-74,461), anti-IRS-1 (sc-559), anti-phosphoenolpyruvate carboxykinase (PEPCK) (sc-32,879), anti-glucokinase (GCK) (sc-7908), and anti-β-actin (sc-19,879) antibodies were purchased from Santa Cruz Biotechnology, Inc. (Dallas, TX, USA). The ECL chemiluminescence kit was purchased from Pierce Manufacturing, Inc. (Bradenton, FL, USA). The NC membranes were purchased from Nanjing Madite Co. (Nanjing, China). The 30% acrylamide, TEMED, XT sample buffer (#161–0791) and XT reducing agent (#161–0792) were purchased from Bio-Rad (Hercules, CA, USA).

### Extraction of the TSCE

Fresh cherry tomatoes (*Lycopersicon esculentum* Mill.) were acquired from the Guangxi Zhuang Valley Agricultural Science and Technology Co., Ltd. (Baise, China) in 2016, and identified by Prof. Yan Liu (Guangxi Institute of Botany). The voucher specimen (LE20160306) was deposited in the Herbarium of Guangxi Institute of Botany, China. The extraction of the TSCE from tomatoes was performed as described previously [12]. In brief, cherry tomatoes (10 kg) were washed and smashed into pulp. The obtained tomato juice was incubated at 50 °C for 2 h with a 0.5% commercial pectinase for enzymatic hydrolysis (Pectinex Ultra SP-L). The mixture was filtered through a 80–100 mesh filter cloth, and then, centrifuged at 3000 rpm/min for 10 min. The supernatant was loaded onto a D-101 macroporous resin column. The column was first washed with water, and, then eluted with 80% ethanol. The 80% ethanol effluent was collected, and TSCE (65 g) was obtained after drying under reduced pressure.

### Isolation and structural characterization of esculeoside A

**Fig. 1:**
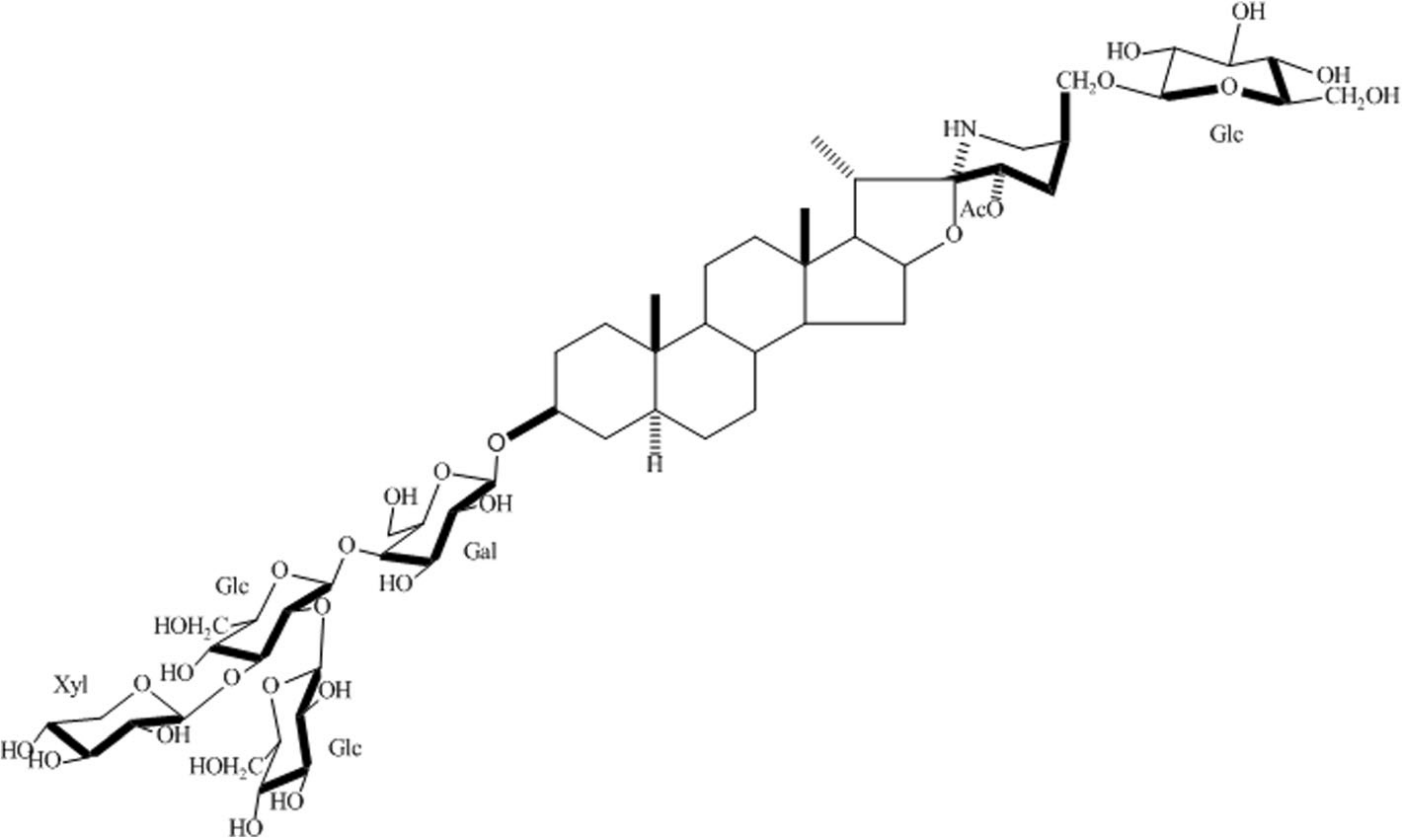
Chemical structure of Esculeoside A

The TSCE (10 g) was dissolved in 30% methanol loaded onto a HP-20ss column, and eluted with a gradient starting from 40% aq. MeOH to 100% MeOH. The 60% eluate was collected, loaded on a Sephadex LH-20 column and eluded with 30% methanol. The procedure yielded 1332 mg of esculeoside A. The structure of esculeoside A was determined by high resolution mass spectrometry and nuclear magnetic resonance spectroscopy. Comparing the data of ^1^H NMR, ^13^C NMR, and high resolution mass spectrometry with reference previous study [17], the structure of esculeoside A was characterized as (23 *S*, 25 *S*) - 23 - acetoxy - 5 *α*, 22 *α N* - 3*β*, 27 - dihydroxyspirosolan 3 - *O* - *β* - lycotetraosyl 27 - *O* - *β* - *D -* glucopyranoside. The chemical structure of esculeoside A is shown in Fig. 1.

### Animals

All animal procedures were previously approved by the Ethical Committee for Animal Research of the Guangxi Zhuang Autonomous Region and Chinese Academy of Science, Guangxi Institute of Botany. Male 4-week-old C57BLKS/Leprdb (*db/db*) mice and wild-type C57BLKS (*db/dm*) mice were purchased from the Model Animal Research Center of Nanjing University.

### Experimental design

The mice were kept at 24 ± 2 °C and 45–50% relative humidity with a 12-h light/12-h dark cycle. The mice were acclimated for 7 days before beginning the experiment. They were provided access to feed and water freely. Eight *db/dm* mice were used in the *db/dm* mouse group and 16 *db/db* mice were randomly divided into 2 groups (8 mice per group): untreated and treated *db/db* mouse groups. Esculeoside A (100 mg/kg) in 0.2 ml/10 g was administered by gavage to the treated *db/db* mouse group. As the vehicle control, distilled water was given to the *db/dm* mouse group and the untreated *db/db* mouse group. All animals were treated once a day for a consecutive 56 days. The mice were weighted every 7 days and the treatment dosage was adjusted according to the body weight of the animals.

At the end of the 56 days, the mice were anesthetized with intraperitoneal injection of 120 mg/kg pentobarbital (manufacturer: HeFei BoMei Biotechnology Co.Ltd., lot number: 110919). After loss of consciousness, blood samples were collected from the abdominal aorta and the mice were sacrificed by cervical dislocation. Blood was placed into a sterile EP tube, centrifuged at 3500 rpm for 10 min at 4 °C, and the serum obtained was stored at − 20 °C. Additionally, the liver were removed, and the liver index was calculated using the following formula: liver index = liver mass (mg)/mice body weight (g). Part of the liver was homogenized using a glass homogenizer, centrifuged at 3500 rpm for 10 min at 4 °C, and the supernatant was stored at − 80 °C. A defined amount of liver tissue was placed in a sterilized frozen tube and stored in liquid nitrogen for western blot analysis. The other part of the liver was stored in a sterilized frozen tube at − 80 °C until hepatic lipid measurement.

### Determination of fasting blood glucose and glucose tolerance

Fasting blood glucose (FBG) was evaluated every 7 days. After 12 h of fasting, 100 mg/kg esculeoside A was administered by gavage to the treated *db/db* mouse group. Distilled water was given by gavage to both the *db/dm* mouse group and the untreated *db/db* mouse group. Two hours later, a blood glucose meter and test strips were used for blood glucose (BG) measurement.

At the end of the 55th day, a glucose tolerance test was conducted. The mice were fasted and treated with esculeoside A (or water as control) as described above, and 2 h later, were intraperitoneally injected with 2.5 g/kg glucose. The blood glucose levels were determined at 0, 0.5, 1, and 2 h later using a blood glucose meter.

### Determination of blood and liver biochemical parameters

Serum levels of TC, TG, ALT, and AST were determined using a semi-automatic biochemical analyzer according to the method described by the manufacturer. Serum levels of INS were determined using an automatic microplate reader according to the manufacturer’s instructions. Hepatic levels of SOD and MDA were determined using a semi-automatic biochemical analyzer according to the method described by the manufacturer. Inflammatory cytokines, including TNF-α, IL-6, and IL-1β were determined using commercial ELISA kits. Liver TC and TG levels were determined using a semi-automatic biochemical analyzer using the tissue TC and TG commercial kit obtained from Beijing ApplyGen Technologies Inc. An accurate amount of liver tissue was weighed and 10 μL/mg of lysis buffer was added. The liver tissue was homogenized using a glass homogenizer, placed in a sterilized EP tube, and allowed to sit for 10 min. The EP tube was then placed in a 70 °C water bath for 10 min. After cooling, the mixture was centrifuged at 2000 rpm for 5 min, the supernatant was collected, and used for tissue TC and TG measurement [18, 19].

### Determination of hepatic insulin signaling-associated protein expression

Liver tissue lysates were prepared using RIPA extraction buffer according to the manufacturer’s instructions. A portion of the supernatant was used for protein concentration determination using the BCA method, and the remaining supernatant was diluted with 4-fold sample buffer, sealed, and heated at 95 °C to denature the protein. The obtained material was then stored at − 80 °C. The protein separation gels with different concentrations were prepared according to the molecular weight of the proteins. Samples with 50 μg of total protein in each sample were loaded and 6% SDS-PAGE gel electrophoresis was carried out using a Cell electrophoresis tank. The proteins on the gel were transferred onto a membrane under a constant current of 0.32 A in ice water bath. After protein transfer was completed, the membrane was blocked by skim milk. Primary antibody was added and incubated overnight at 4 °C. Then, secondary antibody was incubated at 37 °C for 1 h and chemiluminescence determination was carried out after washing. Images were taken using a gel-imager and an enhanced chemiluminescence assay was used for the detection of protein expression. An image processing system was used for semiquantitative analysis of the target bands and a gel analysis software was used for the analysis of the average optical density of each band. The optical density value was used to represent the corresponding protein expression. The expression level of the protein of interest was expressed as a relative value by comparison with the expression level of the internal reference, β-actin [20].

### Statistical analysis

Experimental data were expressed as mean values with corresponding standard errors. One-way analysis of variance (ANOVA) was used for the comparison among multiple samples. Statistical analysis was conducted using SPSS15.0 and *P* < 0.05 was considered as statistically significant.

## Results

### Effects of Esculeoside A on body weight, FBG, and glucose tolerance in *db/db* mice

**Fig. 2:**
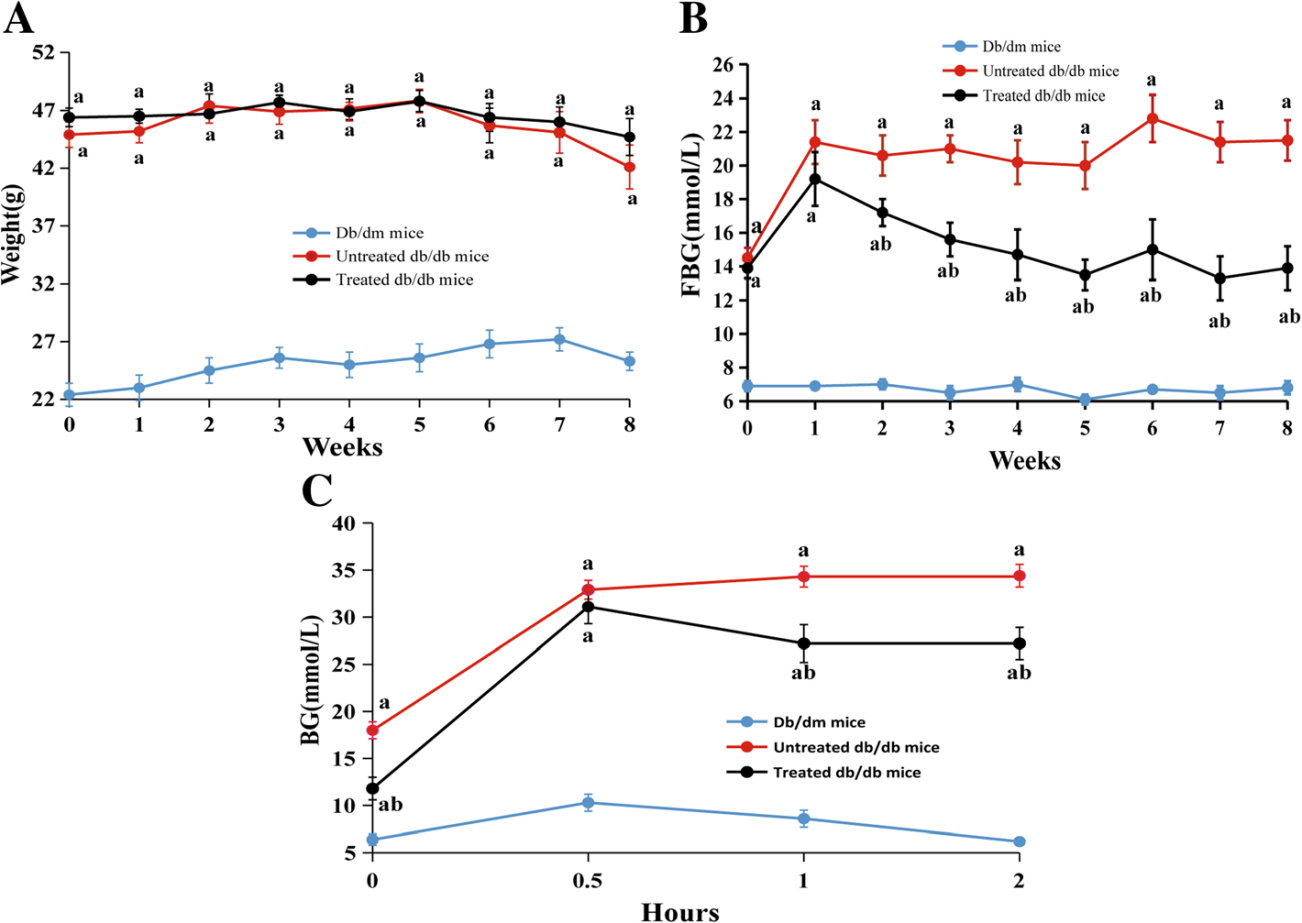
Effects of Esculeoside A on body weight, FBG, and glucose tolerance in *db/db* mice. **A** Body weights, **B** FBG, and **C** glucose tolerance. The results are expressed as mean ± SEM (*n* = 8 per group). Values having different superscripts are significantly different, *P* < 0.05, one-way ANOVA test. a. Statistical difference compared to the *db/dm* mice, b. Statistical difference compared to the untreated *db/db* mice

The changes in the body weight can reflect the growth of the mice and may reflect adverse effects on the body. The body weights of all the mice were stable throughout the experiment (Fig. 2A). Body weights were remarkably higher in the *db/db* mice than the *db/dm* mice. However, there was no statistically significant difference between the esculeoside A-treated and esculeoside A-untreated *db/db* mice.

Although there were no significant changes in body weight in the treated *db/db* mice, the FBG levels was notably decreased after treatment with esculeoside A (Fig. 2B). Reduction in FBG levels occurred after 2 weeks of the treatment, and continued to decline, while that of the control group remained stable during the course of the experiment.

The results of the intraperitoneal glucose tolerance test are shown in Fig. 2C. After intraperitoneal injection of 2.5 g/kg glucose, the BG levels in *db/dm* mice started increasing and reached a peak value after 0.5 h. Thereafter, it gradually decreased and returned to normal levels after 2 h. After intraperitoneal injection of glucose, the BG levels in untreated *db/db* mice exhibited a rapid increase. After 2 h, the BG levels were still increasing. In contrast, the BG levels in treated *db/db* mice rapidly decreased after reaching peak value at 0.5 h. Compared with the untreated *db/db* mice, the BG levels in treated *db/db* mice 20.7% lower 1 h after glucose injection. Two hours after glucose injection, the BG levels were found to be 20.9% lower in treated *db/db* mice.

### Serum and liver analyses 8 weeks after treatment with esculeoside A

As shown in Table 1, the untreated *db/db* mice showed typical type 2 diabetes characteristics, such as elevated levels of insulin and blood lipid, indicating the abnormal metabolisms of lipid as well as insulin resistance. However, esculeoside A administration in *db/db* mice did not significantly affect these parameters.

**Table 1:** Effects of esculeoside A on the biochemical parameters and liver index of *db/db* mice

Item	*Db/dm* mice	Untreated *db/db* mice	Treated *db/db* mice
Serum			
TC (mmol/L)	3.32 ± 0.22	7.05 ± 0.38^a^	6.86 ± 0.87^a^
TG (mmol/L)	0.96 ± 0.08	1.26 ± 0.19^a^	1.20 ± 0.25^a^
INS (mIU/L)	9.0 ± 0.9	70.1 ± 9.5^a^	64.2 ± 12.1^a^
ALT (U/L)	24.3 ± 1.9	59.2 ± 4.8^a^	61.3 ± 7.2^a^
AST (U/L)	49.5 ± 3.1	88.8 ± 7.2^a^	81.5 ± 9.8^a^
TNF-α (ng/L)	95.3 ± 9.8	390.6 ± 23.8^a^	355.1 ± 19.5^b^
IL-6 (ng/L)	33.5 ± 3.6	150.5 ± 14.8^a^	123.8 ± 19.2^a^
IL-1β (ng/L)	6.5 ± 0.7	11.8 ± 1.5^a^	8.5 ± 1.9^a^
Liver			
Liver index (mg/g)	49.58 ± 1.14	57.25 ± 1.57^a^	50.98 ± 1.98^b^
TC (mg/mg prot)	0.16 ± 0.02	0.31 ± 0.04^a^	0.33 ± 0.05^a^
TG (mg//mg prot)	1.67 ± 0.25	5.68 ± 0.61^a^	5.46 ± 0.92^a^
TNF-α (pg/mg prot)	2.38 ± 0.35	4.71 ± 0.49^a^	2.96 ± 0.28^b^
IL-6 (pg/mg prot)	3.12 ± 0.35	12.15 ± 0.78^a^	10.96 ± 1.02^a^
IL-1β (pg/mg prot)	0.77 ± 0.09	2.07 ± 0.22^a^	1.68 ± 0.21^a^
SOD (U/mg prot)	4.61 ± 0.39	4.35 ± 0.42	4.26 ± 0.48
MDA(nmol/mg prot)	2.67 ± 0.31	4.32 ± 0.35^a^	3.11 ± 0.31^b^

The results are expressed as mean ± SEM (*n* = 8 per group). Values having different superscripts are significantly different, *P* < 0.05, one-way ANOVA test. ^a^Statistical difference compared to the *db/dm* mice, ^b^Compared to the untreated *db/db* mice

Examination of the mouse liver found that the liver indices, fat content, and liver damage sensitivity indices, ALT and AST levels, were significantly elevated in the untreated *db/db* mice. After esculeoside A treatment, the liver indexes were significantly decreased. Many inflammatory factors, such as TNF-α, IL-6, and IL-1β are closely associated with liver injury. We used ELISA to detect the levels of TNF-α, IL-6, and IL-1β in serum and liver. The results showed that they were significantly increased in untreated *db/db* mice; however, after esculeoside A administration, only the levels of TNF-α in serum were significantly decreased. We also found the levels of hepatic MDA in untreated *db/db* mice were significantly higher compared to the *db/dm* mice, and esculeoside A treatment significantly prevented the formation of this product of lipid peroxidation.

### Effects of esculeoside A on the expression of proteins involved in glucose uptake

**Fig. 3:**
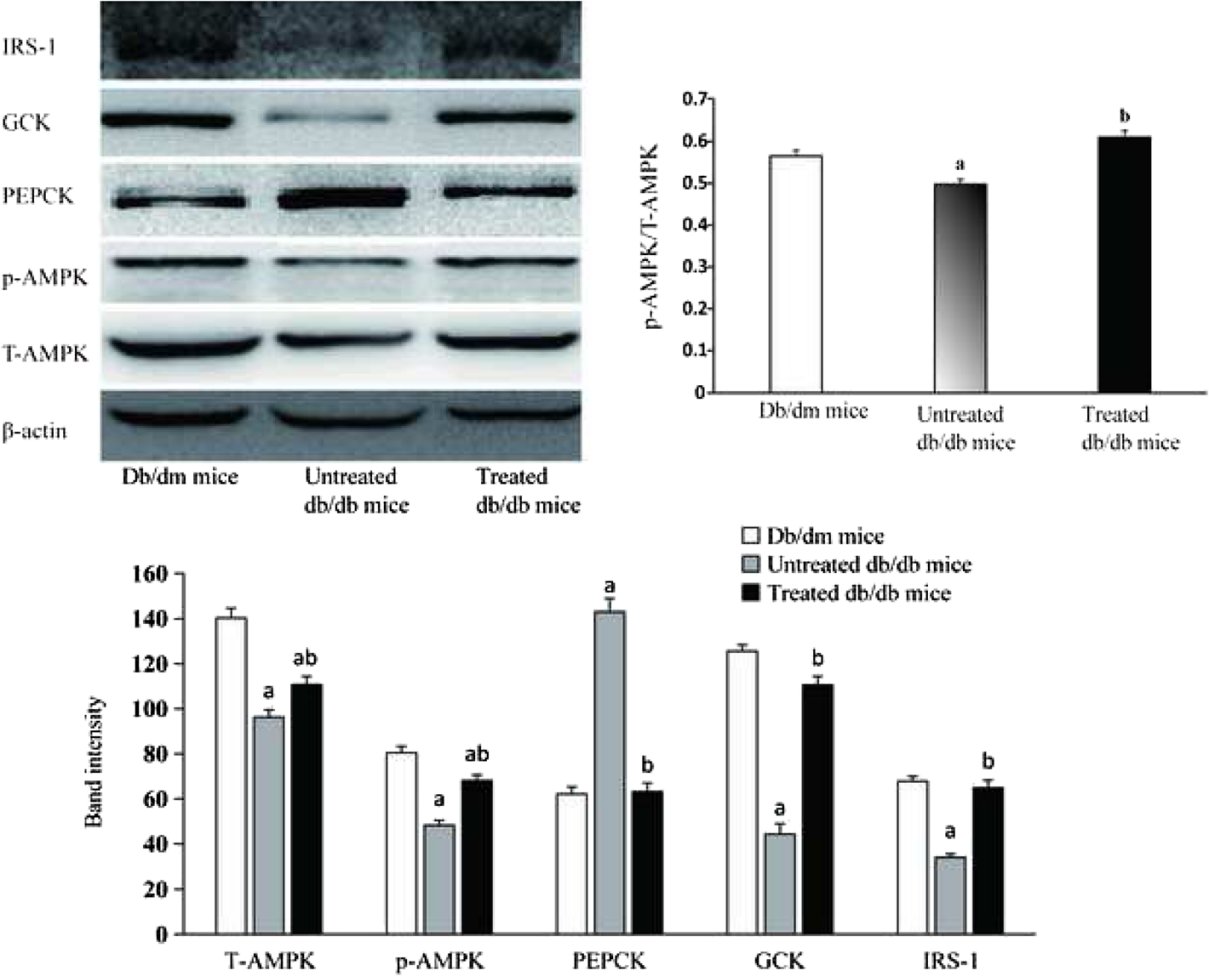
Effects of esculeoside A on the proteins involved in glucose uptake pathway. Values having different superscripts are significantly different, *P* < 0.05, one-way ANOVA test. a. Statistical difference compared to the *db/dm* mice, b. Statistical difference compared to the untreated *db/db* mice

The *db/db* mice were treated with esculeoside A for 56 days, and its effects on the expressions of the proteins associated with glucose uptake were investigated (Fig. 3). After esculeoside A treatment, the hepatic proteins expressions of AMPK, p-AMPK, GCK, and IRS-1 were significantly upregulated. In contrast, the hepatic proteins expression of PEPCK was significantly downregulated after esculeoside A treatment. The hepatic AMPK, p-AMPK, and IRS-1 proteins levels remained low and the hepatic PEPCK protein levels remained high in untreated *db/db* mice. These data suggested that AMPK and IRS-1 pathways contribute to the esculeoside A-mediated downregulation of hepatic glucose production and increase in glucose utilization.

## Discussion

In recent years, the clinical focus has switched to natural products to treat T2DM. Tomatoes have been suggested to be useful in the prevention of diabetes. We extracted the water-soluble saponin compound esculeoside A from *Lycopersicon esculentum* and studied its hypoglycemic effects in experimental type 2 diabetes mice model. The results demonstrated that esculeoside A possessed anti-hyperglycemic properties and the mechanism is promoting AMPK and IRS-1 pathways.

AMPK is considered to be an intracellular “fuel gauge” that plays a vital role in controlling the energy homoeostasis, including the regulation of lipid metabolism, glycogen metabolism, fatty acid oxidation, and BG levels [8, 21]. AMPK can also be used as a therapeutic target for the treatment of metabolism-related diseases [22]. Several drugs have been widely reported to activate AMPK. For instance, metformin significantly enhances AMPK phosphorylation and regulates glycometabolism, and rosiglitazone reduces blood sugar levels and increases AMPK expression [23]. Previous research has shown that the expression of AMPK gene is downregulated in the *db/db* mice [24], and our results were consistent with this finding. The energy metabolism of untreated *db/db* mice was impaired. Maintaining such a state for a long period of time leads to hyperglycemia, hyperlipidemia, and weight gain in untreated experimental animals. After esculeoside A treatment, we observed an upregulation in AMPK expression in the *db/db* mice. The activation of AMPK inhibited two key gluconeogenic enzymes, glucose 6-phosphatase and PEPCK [25]. The major organ for gluconeogenesis is the liver, while PEPCK in liver represents a crucial rate limiting enzyme in the gluconeogenesis pathway, since its transcription level determines the rate of gluconeogenesis [26]. Previous research has indicated that downregulation of PEPCK expression causes reduction in glucose synthesis [27, 28]. In this study, we observed a downregulation of the hepatic PEPCK expression after esculeoside A treatment. This result indicated that the hepatic gluconeogenesis is significantly decreased, eventually leading to decrease BG levels in treated *db/db* mice. These findings suggested that esculeoside A possessed anti-hyperglycemic properties and promoted glucose uptake by activated AMPK pathway in liver. Notably, esculeoside A treatment did not significantly alter TC and TG levels in serum or liver, and did not cause any weight loss in treated *db/db* mice. However, AMPK plays important role in regulating metabolic diseases, such as obesity, diabetes mellitus, etc. It has previously been reported that AMPK also decreased the levels of glucose, cholesterol, and triglycerides, and enhanced fatty acid oxidation [8, 29]. A possible explanation is that esculeoside A activated AMPK pathway was only partially accountable for lowering the blood glucose levels. Furthermore, after esculeoside A treatment, it only increased p-AMPK levels by 33%, but the hepatic PEPCK expression was decreased by 63%. This result suggested that the expression of *PEPCK* gene might be regulated by other factors.

The *db/db* mice are typical hepatic insulin-resistant mice [15]. Decreased insulin sensitivity in the liver can result in increase in glucose production leading to hyperglycemia. Insulin activates metabolism signaling pathways in cells. The signaling pathways regulated by phosphatidylinositol 3-kinase and insulin receptor substrate (IRS) play a vital role in the metabolism of insulin [7]. The two major IRS subtypes known as IRS-1 and IRS-2. They are highly expressed in mice liver. It has previously been reported that both the IRS isoforms have complementary functions in the regulation of hepatic metabolism. IRS-1 gene is more closely associated with hepatic glucose homeostasis, whereas IRS-2 gene is more closely associated with hepatic lipid homeostasis [7]. Studies have shown that Knockdown of IRS-1 gene leads to the upregulation of the expression levels of gluconeogenic enzyme and PEPCK, and the decreased IRS-1 expression is also associated with decreased GCK expression and lead to increase blood glucose [30]. We showed that the hepatic expression of IRS-1 was significantly decreased in untreated *db/db* mice. Abnormity in the insulin signaling pathway is thought to result in increased PEPCK expression and decreased GCK expression. After esculeoside A treatment, the expression level of IRS-1 in liver was restored. As mentioned earlier, the hepatic PEPCK expression in the treated *db/db* mice was decreased. GCK is mainly expressed in the liver and catalyzes cell glucose phosphorylation, and is responsible for glucose homeostasis [31]. Loss of GCK activity leads to diabetes in humans and animals [32]. Our study showed that the expression of GCK in the treated *db/db* mice was 150% higher than in the untreated *db/db* mice. The hepatic PEPCK expression was decreased and GCK expression was increased, both contributing to reduction of BG levels in treated *db/db* mice. Therefore, we speculated that esculeoside A could regulate the function of insulin and promote insulin signal transduction.

In the present study, we showed that glucose tolerance in untreated *db/db* mice was negatively affected. Impaired glucose tolerance was mainly due to insulin resistance in muscles and fat tissues [33, 34], as characterized by reduced insulin-induced muscle and fat absorption of glucose, resulting in reduced glucose utilization and increased postprandial blood sugar. Due to the ability of esculeoside A to improve the impaired glucose tolerance in treated *db/db* mice, we speculated that it is partly accountable for the treatment type 2 diabetes by improving the sensitivity of muscles or fat to insulin, thereby increasing their glucose uptake. In addition, in order to evaluate the toxic side effects of esculeoside A in the liver, the liver indices, the AST and ALT were evaluated. The results showed that esculeoside A treatment reduced the liver swelling, but did not change the ALT and AST levels. In addition, we found that esculeoside A restored the MDA and TNF-α levels in the liver. These results indicated that esculeoside A might exhibit protective effects on the liver.

Tomato is considered as a healthy food with very low glycemic index, which makes it a healthy food for diabetics [35]. Indeed, tomato consumption has been associated with a reduced risk of chronic non-communicable diseases, including diabetes [9]. However, the epidemiologic studies on the role of tomatoes in prevention of T2DM are limited. It is generally believed that lycopene represents the main bioactive compound in tomatoes. Lycopene is a powerful free radical scavenger and tomatoes are a rich source of lycopene. Recent studies have shown that antioxidants exhibit protective effects against the development of diabetes [36]. A few previous studies have assessed the effects of lycopene on BG levels, lycopene supplementation or lycopene-containing foods appear to exhibit beneficial effects on insulin resistance or BG levels in experimental type 2 diabetes model [10, 37, 38]. However, direct evidence with respect to the beneficial effects of lycopene on BG levels is still lacking; moreover, the epidemiologic studies on the association between lycopene or lycopene-containing foods and T2DM are scarce. Many studies have suggested that lycopene may not have a role in the prevention of T2DM [35, 39, 40]. In present study, we demonstrated that tomato main compound esculeoside A possessed anti-hyperglycemic properties, which indicated that the effective component of tomato responsible for hypoglycemic effects is esculeoside A. As far as we know, there is no published study for dietary tomatoes decreased blood sugar in humans. The reason might be that the level of Esculeoside A is very low in dietary tomatoes. In our study, esculeoside A accounted for 0.021% of dry weight of raw cherry tomatoes; 100 mg/kg esculeoside A was administered by gavage to the *db/db* mice which reduced the FBG levels. According to the previous literature [41] and the results of this study, if the mouse data is extrapolated to humans, a person must consume 1802 g of raw tomatoes to obtain enough esculeoside A that could provide adequate beneficial effects by lowering blood glucose. In previous epidemiologic studies, volunteers were not request to consume such high quantity of tomatoes every day. Our findings suggested a possible usefulness of esculeoside A (or TSCE) as a functional supplement for diabetes treatment; however, the possible beneficial effects of esculeoside A in human diabetes need to be further studied.

## Conclusions

These findings suggested that esculeoside A has the potential of alleviating the metabolic abnormalities in diabetic mice via regulation of AMPK/IRS-1 pathway. Our findings also supported a potential role of esculeoside A as a functional supplement for diabetes treatment.

## Data Availability

The datasets analyzed during the current study are available from the corresponding author on reasonable request.

## Abbreviations

ALT: Alanine aminotransferase
AMPK: Amp-activated protein kinase
AST: Aspartate aminotransferase
FBG: Fasting blood glucose
GCK: Glucokinase
IL-1β: Interleukin-1β
IL-6: Interleukin-6
INS: Insulin
IPGTT: Intraperitoneal glucose tolerance test
IRS-1: Insulin receptor substrate-1
IRS-2: Insulin receptor substrate-2
MDA: Malondialdehyde
p-AMPK: phosphorylated Amp-activated protein kinase
PEPCK: Phosphoenolpyruvate carboxykinase
SOD: Superoxide dismutase
TC: Total cholesterol
TG: Triglycerides
TNF-α: Tumor necrosis factor
